# Prevalence of dental caries among children attending public versus private schools: A cross-sectional comparative analysis

**DOI:** 10.6026/973206300214668

**Published:** 2025-12-15

**Authors:** Anju Singh, Konark Singh

**Affiliations:** 1 Department of Dentistry, Patna Medical College and Hospital, Patna, Bihar, India; 2 Department of Conservative Dentistry and Endodontics, Patna Dental College and Hospital, Patna, Bihar, India

**Keywords:** Dental caries, socioeconomic status, school children, oral health disparities, DMFT index, public health dentistry

## Abstract

Dental caries represents a major global public health challenge, particularly among children in low-socioeconomic settings, where poor oral hygiene, high sugar intake, and limited access to dental care exacerbate prevalence and severity, leading to pain, infection, and long- term oral health disparities. Hence, a cross-sectional comparative study was conducted on 812 schoolchildren aged 8-10 years, selected via a multistage random sampling method from public (n=405) and private (n=407) schools. Data were collected through a clinical oral examination using the World Health Organization (WHO) 2013 criteria to determine the Decayed, Missing and Filled Teeth index for both primary (DMFT) and permanent (DMFT) dentition. A pre-tested questionnaire was used to gather information on oral hygiene practices and dietary habits. A significant disparity in dental caries prevalence and experience exists between children attending public and private schools, with the burden being substantially higher in the public school population. Thus, we show the influence of socioeconomic factors on children's oral health and highlight the urgent need for targeted, school-based public health interventions in underprivileged communities.

## Background:

Dental caries is a multifactorial, biofilm-mediated disease characterized by the demineralization of tooth hard tissues and it remains a major global public health problem [1]. Despite significant advances in prevention and treatment, it is the most common chronic disease of childhood, affecting a substantial proportion of children worldwide [2]. The consequences of untreated dental caries in children are far-reaching, including pain, infection, and difficulty in mastication, nutritional impairment, poor school performance and a reduced quality of life [3]. The economic burden of treating this entirely preventable disease is also immense, placing a significant strain on healthcare systems, particularly in low- and middle-income countries [4]. The distribution of dental caries is not uniform across populations; it exhibits a strong social gradient, with a disproportionately higher burden on individuals from lower socioeconomic backgrounds [5]. Socioeconomic status (SES) encompasses various factors such as parental income, education level and occupation, which collectively influence health-related knowledge, attitudes, behaviors and access to healthcare services [6]. For instance, lower SES is often associated with diets high in refined carbohydrates, suboptimal oral hygiene practices and limited access to preventive and restorative dental care [7, 8]. In many societies, the type of school a child attends-public (government-funded) or private-often serves as a reliable proxy for their family's socioeconomic standing [9]. Children enrolled in private schools typically come from families with higher income and educational attainment, whereas public schools predominantly serve children from lower- and middle-income families. This dichotomy provides a natural setting to investigate the impact of socioeconomic disparities on health outcomes. Several international studies have documented a higher prevalence of dental caries among children in public schools compared to their counterparts in private schools [10, 11]. For example, a study in Brazil found that students from public schools had a significantly higher DMFT index and greater unmet treatment needs [12]. While the general association between SES and dental caries is well-established, there is a continuous need for current, region-specific data to inform local public health policies and interventions. Rapid urbanization and changing lifestyle patterns in countries like India necessitate on-going surveillance of oral health trends. Most existing studies either focus on a single school type or do not adequately control for confounding factors. A direct, robust comparison of caries prevalence and associated risk factors between public and private school systems within the same geographic area can provide clear, actionable evidence of existing health inequities. Therefore, it is of interest to bridge this gap by conducting a comprehensive comparative assessment. Therefore, it is of interest to evaluate and compare the prevalence and severity of dental caries in 8-10-year-old children attending public and private schools in a major metropolitan area.

## Materials and Methods:

## Study design and setting:

This was a cross-sectional, comparative epidemiological study conducted between September 2023 and March 2024. The study was designed to compare two distinct populations of schoolchildren: those attending government-funded public schools and those attending fee-paying private schools.

## Sample size calculation and sampling technique:

The sample size was calculated using a standard formula for comparing two proportions. Based on a previous pilot study and literature review, the anticipated caries prevalence was estimated to be 70% in public schools and 50% in private schools. To achieve a study power of 90% and a significance level (alpha) of 0.05, a minimum sample size of 385 students per group was required. To account for potential non-responses or incomplete data, the target sample size was inflated by 5%, resulting in a final target of approximately 405 students per group. A multistage stratified random sampling technique was employed. First, the city was divided into four administrative zones. From each zone, two public schools and two private schools were randomly selected from a comprehensive list provided by the local education authority. From each selected school, two classes from Grades 3, 4 and 5 (corresponding to the 8-10 year age group) were randomly chosen. Finally, from each selected class, a list of all eligible students was prepared and 18-20 students were randomly selected using a random number table.

## Inclusion and exclusion criteria:

Students were included if they were aged 8-10 years, were present in the class on the day of the examination and provided written informed consent from their parents/guardians and verbal assent themselves. Children with severe systemic diseases, developmental anomalies, or those currently undergoing orthodontic treatment were excluded from the study.

## Ethical considerations:

Ethical clearance for the study was obtained from the Institutional Ethics Committee of the National Institute of Dental Sciences (Ref: NIDS/IEC/2022/048). Official permission was secured from the municipal education authorities and the principals of the selected schools. The study's purpose and procedures were explained to the parents/guardians and written informed consent was obtained.

## Data collection:

Data were collected using two methods: a clinical oral examination and a structured questionnaire.

## Clinical examination:

All examinations were conducted within the school premises in a well-lit area with students seated on a regular chair. A team of two trained and calibrated dental examiners performed the examinations using sterilized plane mouth mirrors and WHO Community Periodontal Index (CPI) probes under a portable LED headlight. Dental caries was diagnosed according to the WHO criteria (2013) and the findings were recorded on a specially designed proforma. The Decayed, Missing and Filled Teeth indices for both primary (dmft) and permanent (DMFT) teeth were recorded for each child. The "d" and "D" components represented untreated caries. Before the main study, the examiners underwent a calibration exercise on a group of 25 children not included in the final sample. The inter-examiner reliability was assessed using the Kappa statistic, which was found to be 0.89, indicating excellent agreement.

## Questionnaire:

A pre-tested, closed-ended questionnaire was administered to each child through a face-to-face interview by trained research assistants to ensure comprehension.

The questionnaire collected information on:

[1] Demographics: Age, gender.

[2] Socioeconomic Status: Parental education and occupation (used to classify SES based on the modified Kuppuswamy scale).

[3] Oral Hygiene Practices: Frequency of toothbrushing, materials used for cleaning.

[4] Dietary Habits: Frequency of consumption of sugary foods and beverages (e.g., sweets, chocolates, soft drinks), categorized as 'low' (≤1 time/week), 'moderate' (2-4 times/week), or 'high' (≥5 times/week).

## Statistical analysis:

All collected data were entered into Microsoft Excel and analyzed using the Statistical Package for the Social Sciences (SPSS) Version 26.0 (IBM Corp., Armonk, NY). Descriptive statistics including mean, standard deviation (SD), frequencies and percentages were used to summarize the data. The Chi-square test was used to compare categorical variables (e.g., caries prevalence, oral hygiene habits) between the two school groups. The independent samples t-test was used to compare the mean dmft and DMFT scores. A p-value of < 0.05 was considered statistically significant for all tests.

## Results:

A total of 812 children (405 from public schools, 407 from private schools) participated in the study. The mean age of the participants was 9.1 ± 0.8 years. The gender distribution was comparable between the two groups (p=0.712). As expected, there was a significant difference in the socioeconomic status between the groups. A vast majority of children in private schools (85.5%) belonged to the upper or upper-middle SES categories, whereas most children in public schools (79.3%) belonged to the lower-middle or lower SES categories (p < 0.001). This confirms that school type was an effective proxy for SES in this study population (Table 1 - see PDF). The prevalence of dental caries was significantly higher in children attending public schools compared to those in private schools. In the public school group, 303 out of 405 children (74.8%) had at least one carious lesion (dmft/DMFT > 0), whereas in the private school group, 186 out of 407 children (45.7%) were affected (p < 0.001). The severity of caries, measured by mean dmft and DMFT scores, also showed a significant disparity (Table 2 - see PDF). The mean dmft score for public school children was more than double that of private school children (3.8 ± 2.1 vs. 1.5 ± 1.2; p < 0.001). A similar trend was observed for the permanent dentition, with a mean DMFT of 1.2 ± 0.9 in the public school group versus 0.5 ± 0.6 in the private school group (p < 0.001). Furthermore, the analysis of the components revealed that the "decayed" (D/d) component constituted the largest proportion of the total index in the public school group, indicating a high level of unmet treatment needs. Significant differences were observed in the self-reported oral health behaviors between the two groups (Table 3 - see PDF). Only 35.3% of children from public schools reported brushing their teeth twice daily, compared to 78.1% of children from private schools (p < 0.001). Regarding dietary habits, 62.5% of children in public schools reported high-frequency sugar consumption (≥5 times/week), which was significantly higher than the 28.7% reported by children in private schools (p < 0.001).

## Discussion:

The findings of this large-scale, cross-sectional study clearly demonstrate a profound disparity in the prevalence and severity of dental caries between children attending public and private schools in an urban Indian setting. Children from public schools exhibited a significantly higher burden of disease, with higher caries prevalence, greater mean dmft/DMFT scores and substantially more untreated decay. These results are consistent with the global body of evidence that positions socioeconomic status as a primary determinant of oral health outcomes in children [13, 14]. The observed caries prevalence of 74.8% in public school children is alarming and points to a significant public health challenge. This finding aligns with studies conducted in similar demographic settings in India and other developing nations, where caries rates in underprivileged populations often exceed 70% [15]. In stark contrast, the 45.7% prevalence in private school children, while still considerable, reflects the protective effect of higher socioeconomic status. Our study successfully established that school type was a strong proxy for SES, with parental education and income levels differing significantly between the two groups. This socioeconomic differential is the likely root cause of the observed health disparity [6]. The mechanisms through which SES influences caries risk are multifaceted and were partly elucidated by our secondary findings on oral health behaviors. Children from private schools reported significantly better oral hygiene practices (more frequent tooth brushing) and healthier dietary habits (less frequent sugar consumption). This is likely attributable to higher parental health literacy, greater awareness of preventive health measures and the financial ability to afford healthier food options and dental products [16]. Conversely, children from lower SES backgrounds often face barriers such as lack of knowledge, affordability issues and exposure to a community environment where cariogenic diets are normative [8]. One of the most critical findings of this study is the disparity in unmet treatment needs. In the public school group, the "decayed" (d/D) component accounted for the vast majority of the total daft/DMFT score. This indicates that while the disease is rampant, access to or utilization of restorative dental care is extremely low. In contrast, the "filled" (f/F) component was significantly higher in the private school group, suggesting that these children not only have a lower disease incidence but also better access to dental services when disease does occur [12]. This "inverse care law," where those with the greatest need have the least access to care, is a well-documented phenomenon in health services research and is starkly evident in our findings [17]. Our study's results echo those from international research. For instance, Dettori *et al.* in Italy found that children from lower SES backgrounds had a significantly higher DMFT and a higher likelihood of having untreated caries [18]. Similarly, a study in the United States by Bansal *et al.* highlighted that children from low-income families are twice as likely to have untreated decay as their more affluent peers [19]. The consistency of these findings across different cultural and healthcare contexts underscores the universal nature of socioeconomic health inequalities. This study has several strengths, including its large sample size, the use of a robust multistage random sampling technique to ensure representativeness and the adherence to standardized WHO diagnostic criteria with calibrated examiners. However, some limitations should be acknowledged. Firstly, the cross-sectional design precludes any inference of causality between risk factors and disease. Secondly, data on oral hygiene and dietary habits were self-reported by children, which may be subject to recall bias or social desirability bias. Finally, while conducted in a large metropolitan area, the findings may not be generalizable to rural populations, where the determinants of health may differ. The public health implications of these findings are significant. The vast disparity in oral health highlights the failure of a "one-size-fits-all" approach to child oral health promotion. There is an urgent need for targeted, school-based interventions specifically for public schools. These programs should be comprehensive, including oral health education for children and parents, supervised tooth brushing drills, application of topical fluorides and the provision of basic screening and referral services [20].

## Conclusion:

We show significant disparity in dental caries experience between children attending public and private schools. The burden of disease is disproportionately borne by children from public schools, who reflect a lower socioeconomic stratum. This inequality is driven by differences in oral health behaviors and, most critically, in access to and utilization of dental care.
